# A novel peptide that disrupts the Lck-IP3R protein-protein interaction induces widespread cell death in leukemia and lymphoma

**DOI:** 10.21203/rs.3.rs-2436910/v1

**Published:** 2023-01-09

**Authors:** Michael Harr, Andrew Lavik, Karen McColl, Fei Zhong, Ben Haberer, Khadijah Aldabbagh, Vivien Yee, Clark W. Distelhorst

**Affiliations:** Case Western Reserve University; Case Western Reserve University; Case Western Reserve University; Case Western Reserve University; Case Western Reserve University; Case Western Reserve University; Case Western Reserve University; Case Western Reserve University

## Abstract

There is increasing evidence that the T-cell protein, Lck, is involved in the pathogenesis of chronic lymphocytic leukemia (CLL) as well as other leukemias and lymphomas. We previously discovered that Lck binds to domain 5 of inositol 1,4,5-trisphosphate receptors (IP3R) to regulate Ca2+ homeostasis. Using bioinformatics, we targeted a region within domain 5 of IP3R-1 predicted to facilitate protein-protein interactions (PPIs). We generated a synthetic 21 amino acid peptide, KKRMDLVLELKNNASKLLLAI, which constitutes a domain 5 sub-domain (D5SD) of IP3R-1 that specifically binds Lck via its SH2 domain. With the addition of an HIV-TAT sequence to enable cell permeability of D5SD peptide, we observed wide-spread, Ca2+-dependent, cell killing of hematological cancer cells when the Lck-IP3R PPI was disrupted by TAT-D5SD. All cell lines and primary cells were sensitive to D5SD peptide, but malignant T-cells were less sensitive compared with B-cell or myeloid malignancies. Mining of RNA-seq data showed that LCK was expressed in primary diffuse large B-cell lymphoma (DLBCL) as well as acute myeloid leukemia (AML). In fact, LCK shows a similar pattern of expression as many well-characterized AML oncogenes and is part of a protein interactome that includes FLT3-ITD, Notch-1, and Kit. Consistent with these findings, our data suggest that the Lck-IP3R PPI may protect malignant hematopoietic cells from death. Importantly, TAT-D5SD showed no cytotoxicity in three different non-hematopoietic cell lines; thus its ability to induce cell death appears specific to hematopoietic cells. Together, these data show that a peptide designed to disrupt the Lck-IP3R PPI has a wide range of pre-clinical activity in leukemia and lymphoma.

## Introduction

The lymphocyte-specific protein tyrosine kinase (Lck) is a member of the Src family of protein tyrosine kinases first identified in the 1980s (1, 2). Since then, the function of Lck has been extensively investigated and many mechanistic insights into the regulation of its activity have been revealed. However, because Lck is primarily expressed in T cells, few reports have investigated the role of Lck in malignant B cells or other cells of hematopoietic origin (3).

Recent studies are beginning to elucidate how the alteration in expression and/or activity of Lck may result in pathological conditions like chronic lymphocytic leukemia (CLL) and other blood cancers (3). We among other groups have shown that Lck is aberrantly expressed in CLL cells compared with normal B cells (4–11). CLL cells with high expression of Lck show elevated BCR signaling capacity, cell survival and protection from glucocorticoid-induced apoptosis (4, 9, 10). Moreover, selective inhibition of Lck is sufficient to block BCR signaling in CLL (9). While it is not known how Lck regulates downstream BCR signaling, it may be required for the activation of PLCγ (11), which is responsible for the generation of critical second messengers such as IP_3_, DAG, and subsequent Ca^2+^ signaling mediated by IP_3_R (12).

Using a combination of biochemical and bioinformatic approaches, our laboratory discovered that Lck binds to IP_3_R at a 21 amino acid region (2078–2098) KKRMDLVLELKNNASKLLLAI (13). In T cells, the Lck-IP_3_R protein-protein interaction (PPI) regulates the pattern of Ca^2+^ flux by a mechanism that is independent of IP_3_. A synthetic version of KKRMDLVLELKNNASKLLLAI, referred to as domain 5 sub-domain (D5SD), inhibited binding of Lck to IP_3_R-1 and shifted the pattern of Ca^2+^ signaling after strong T-cell receptor activation. In this study, we hypothesized that D5SD peptide would affect cell proliferation and/or death in hematological malignancies by sequestering Lck or potentially other proteins that can bind to domain 5 of IP_3_Rs. Because Lck has emerged as a druggable target for many cancers (3), these data have important clinical implications. CLL, mantle cell lymphomas, T-cell neoplasms, and many lymphomas of germinal center origin have been found to express Lck (4–11, 14). Recent studies have also linked Lck to the pathogenesis of AML, suggesting that it may function as an oncogene in myeloid malignancies (15, 16).

## Results

### Design of D5SD Peptide

**Figure 1:**
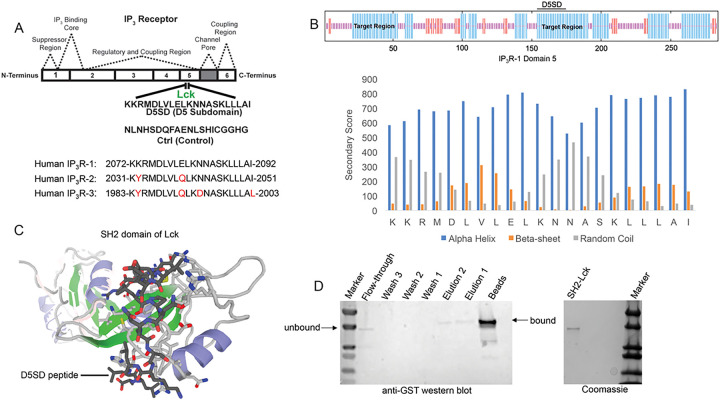
Design and synthesis of D5SD peptide. **(A)** Lck interacts with IP_3_R-1 (13). Shown is the 21 amino acid sequence (2078–2098), KKRMDLVLELKNNASKLLLAI, within Domain 5 of IP_3_R-1 where Lck interacts. This amino acid sequence corresponds to the D5SD peptide used in subsequent experiments. The control peptide sequence (NLNHSDQFAENLSHICGGHG) was also derived from Domain 5 of IP_3_R-1, but the sequence was scrambled so that it had no significant homology to any other mammalian protein. Amino acid sequences of IP_3_R-2 and IP_3_R-3 show significant homology to IP_3_R-1 and are shown for comparison. **(B)** Top, schematic of domain 5 of IP_3_R-1 showing target regions with the highest probability of forming alpha-helices (blue). Bottom, specific secondary structure elements (alpha-helices vs beta sheets and random coils) within the D5SD amino acid sequence as assessed by the GOR IV algorithm. A higher score denotes increased probability for each state of secondary structure. **(C)** Molecular modeling of D5SD peptide (dark gray) bound to the SH2 domain of Lck. Modeling was performed using GalaxyPepDock, a web-based supercomputer that uses similarity and interaction scores followed by energy-based optimization to predict peptide docking sites. **(D)** Left, western blot detecting the SH2 domain of Lck (GST-tagged) binding to biotin-labelled D5SD peptide immobilized on streptavidin-coated beads. The beads and elution lanes show bound GST-SH2. The flow-through lane shows a band for unbound GST-SH2. Right, visualization of the purified GST-tagged Lck protein (unbound) on an SDS gel stained with Coomassie Brilliant Blue.

As noted, Lck binds IP_3_R at Domain 5 which corresponds to a 21 amino acid sequence from IP_3_R-1 (13). A synthetic peptide, domain 5 sub-domain (D5SD), was generated to competitively bind Lck and displace it from IP_3_R (Fig. 1A). Moreover, we designed and synthesized a scrambled peptide as a control, also from the IP_3_R-1 sequence, that had no significant homology to any mammalian protein (Fig. 1A). When generating the D5SD peptide, the GOR IV algorithm was utilized to predict the secondary structure of domain 5 within IP_3_R-1 (17) and was validated by an additional algorithm developed by Rost and Sander (18). As shown in Fig. 1B (top), there were two target regions (blue) within domain 5 of IP_3_R-1 that were strongly predicted to form alpha helices. We chose the 21 amino acid sequence starting at position 155, because the two lysine residues at the N terminus facilitate protein-protein interactions (PPIs) (18, 19). As shown in Fig. 1B (bottom), this region is predicted to form alpha helices vs beta sheets or random coils. While our experiments focused on using the human IP_3_R-1 fragment, IP_3_R-2 and IP_3_R-3 fragments are shown for comparison because they are highly homologous sequences (Fig. 1A).

### D5SD Peptide Binds to the SH2 domain of Lck

To visualize how D5SD peptide might bind Lck, we used a computational approach that models PPIs with a high degree of accuracy. Using a 3D molecular structure of the Lck SH2 domain from the protein data bank (PDB), we were able to predict where D5SD peptide would bind (Fig. 1C). The model shown utilizes similarity and interaction scores followed by an energy-based refinement process to determine the most optimal molecular flexibility for the PPI. To validate this model, we assessed whether biotin-labelled D5SD peptide would bind a puri ed GST-tagged SH2 domain from Lck. Pull-down experiments revealed a small fraction of unbound SH2 in the flow-through at ~ 36kDa. However, the majority of SH2 was bound to immobilized D5SD (note the shift in migration of the SH2 band), while a lower fraction of D5SD-bound SH2 was observed in the eluate (Fig. 1D). These data suggest that D5SD peptide binds Lck within the SH2 domain.

### D5SD Peptide Disrupts the Lck-IP_3_R PPI

**Figure 2:**
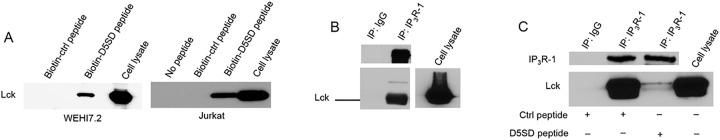
D5SD peptide binds Lck and disrupts the Lck-IP_3_R PPI. **(A)** Western blot detecting endogenous Lck protein pulled down from WEHI7.2 (murine) and Jurkat (human) cell lysates by biotin-labelled D5SD peptide compared with biotin-labelled control peptide. **(B)** Co-immunoprecipitation demonstrating the Lck-IP_3_R-1 PPI in Jurkat T cells. **(C)** Co-immunoprecipitation showing that incubation of WEHI7.2 cell lysate with D5SD peptide inhibits the Lck-IP_3_R-1 PPI. Results were confirmed in multiple independent experiments.

To confirm that full-length Lck binds to D5SD peptide, we incubated biotin-labelled peptides with lysates from WEHI7.2 and Jurkat T-cell lines. Indeed, biotin-labelled D5SD peptide binds Lck in lysates from both cell lines, whereas the control peptide does not (Fig. 2A). Because our previous work on the Lck-IP_3_R PPI had been conducted in WEHI7.2 murine cells, we wanted to con rm that the endogenous Lck-IP_3_R PPI was also present in human cells; Fig. 2B indicates that this interaction also naturally occurs in jurkat (human) T cells (Fig. 2B). Importantly, Fig. 2C shows that D5SD peptide markedly disrupts the Lck-IP_3_R PPI. Notably, in B cells, where the Src kinase Lyn is often more abundant than Lck, we did not observe an interaction between Lyn and IP_3_R **(Fig S1).** This suggests that D5SD peptide may preferentially bind Lck over other Src family kinases.

### Lck Helps Malignant Hematopoietic Cells Maintain Viability

Next, we hypothesized that D5SD peptide would induce cell death in lymphoid malignancies due to its ability to regulate Ca^2+^ signals in T-cells (13). In order to maximize cellular uptake of peptides in subsequent experiments, HIV-TAT sequences were added to both D5SD and control peptides **(Fig S2A).** Importantly, D5SD peptide without the TAT sequence had no effect on cell viability compared with an untreated control **(Fig S2B).** Thus, the effect of TAT-D5SD peptide on cell viability was measured in various hematological malignancies, including lymphoid and myeloid lineages.

**Figure 3:**
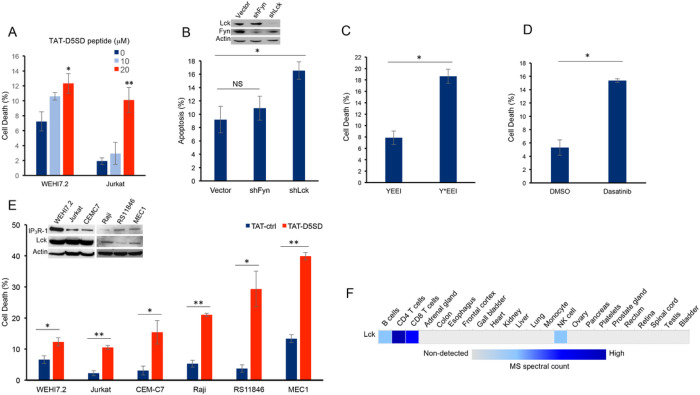
D5SD peptide induces cell death in lymphoid malignancies. **(A)** WEHI7.2 and Jurkat T cells were treated with 0, 10, or 20 mM TAT-D5SD for 24 h and cell death was measured by Trypan Blue dye exclusion. **(B)** WEHI7.2 cells transduced with lentiviral vectors encoding shRNAs for Lck and Fyn. Apoptosis was measured by staining cells with annexin-V and propidium iodide and analyzed by ow cytometry. **(C)** Primary CLL cells were treated with H_2_O-soluble phosphorylated EGQY*EEIP peptide (200 nM) for 24 h to bind the SH2 domain of Lck and inhibit its catalytic activity (non-phosphorylated EGQYEEIP was used as a control); both peptides are cell-permeable and do not require a TAT sequence. **(D)** Primary CLL cells were treated with dasatinib (100 nM) for 24 hours to inhibit Src kinase activity. **(E)** Malignant T-cell lines (WEHI7.2, Jurkat, CEMC7) and B-cell lines (Raji, RS11846, MEC1) were treated with TAT-D5SD or TAT-ctrl (20 μM) for 24 h. The expression of Lck and IP_3_R-1 is shown for each cell line in the inset. **(F)** Heat map showing quantification of Lck by high resolution Fourier mass spectrometry (publicly available from the human proteome map) in hematopoietic cells vs other tissues. Results were confirmed in multiple independent experiments. Cell death was measured by Trypan Blue dye exclusion unless otherwise indicated. Bars represent the mean ± SEM. A student’s T-test was used to determine statistical significance; *P<0.05; **P<0.01.

We initially tested TAT-D5SD in the two malignant T-cell lines that were used in the previously described biochemical assays. In WEHI7.2 and Jurkat T cells, we observed a modest, yet significant induction of cell death in both cell lines with TAT-D5SD (20 μM) after 24 hours (Fig. 3A). To determine if Lck was important for cell survival, we created a WEHI7.2 cell line where Lck was constitutively knocked-down by lentiviral-mediated shRNA. Here we show a significantly higher level of apoptosis in cells that do not express Lck, but do express Fyn (Fig. 3B). Both Lck and Fyn are highly expressed in T cells, whereas Lyn is not (20); importantly, shNRA-mediated silencing of Fyn had no significant effect on the percentage of apoptotic cells (Fig. 3B). These data suggest a unique role for Lck in regulating cell death.

We also tested whether selectively targeting the SH2 domain of Lck affects cell viability in B-CLL cells which co-express Lck and Lyn. Treatment of CLL cells with the cell-permeable phospho-peptide EGQY*EEIP has been shown to preferentially bind the SH2 domain of Lck vs Lyn due to the specificity of the YEEI sequence, which prevents activation of Lck’s catalytic domain (21). We found that the phosphorylated form of the peptide (Y*EEI), selectively targeting the SH2 domain of Lck, more than doubled the level of cell death in CLL cells (Fig. 3C). Moreover, a similar level of cell death was observed after pharmacological inhibition of Lck with the pan-Src inhibitor dasatinib (Fig. 3D). While dasatinib inhibits several Src family members, it has a higher selectivity for Lck and Src compared with Lyn and Fyn (22). Taken together, these data suggest that Lck, in part, helps maintain the survival of malignant hematopoietic cells.

Based on these data, we tested an additional T-cell malignancy and three B-cell malignancies to determine the effects of TAT-D5SD peptide on cell viability. Specific details of each cell line are shown in Table 1. All three malignant T cell lines were less sensitive to TAT-D5SD compared with malignant B-cell models (Fig. 3E). MEC1, a CLL cell line, was among the most sensitive to TAT-D5SD and showed 40% cell death after 24 hours of treatment. All cell lines expressed IP_3_R-1 to varying degrees (Fig. 3E, inset). While most B cells co-express Lyn and Lck, western blot analysis confirmed that Lck was expressed in Raji, RS11846, and MEC1 cells (Fig. 3E, inset). This is consistent with other studies which have shown Lck to be expressed in a number of B cell malignancies, including CLL and several types of B-cell lymphoma (4–11, 14). In fact, Lck protein levels are quantifiable by ultra-sensitive mass spectrometry in normal B cells (low expression) and T cells (high expression), but generally not expressed in non-hematopoietic cells (Fig. 3F). Importantly, we did not observe any significant effects on cell viability in non-hematopoietic cell lines such as NIH 3T3, NL20, and HEK-293 **(Fig S3)**.

### TAT-D5SD Peptide Induces Cell Death in Primary CLL Cells by a Ca^2+−^Dependent Mechanism

**Figure 4:**
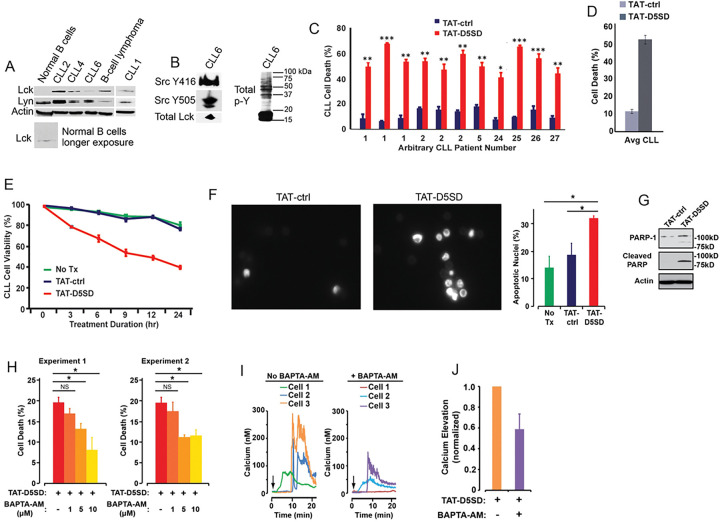
D5SD peptide induces Ca^2+-^dependent killing of CLL cells. **(A)** Lck and Lyn are variably expressed in CLL and B-cell lymphoma. Western blot showing Lck and Lyn expression from primary peripheral blood lymphocytes isolated from patients with CLL (one patient with marginal zone lymphoma) or normal CD19+ B cells. Each CLL sample was obtained with consent and IRB approval from University Hospitals Cleveland Medical Cancer and immediately processed. **(B)** Total and phosphorylated Lck from CLL6 isolated from an independent blood-draw and lysed in SDS sample buffer. Phospho-antibodies are specific for both phosphorylation sites, but do not differentiate between different Src family kinases. A blot showing total tyrosine phosphorylation is shown. **(C)** Primary human CLL cells were treated with TAT-D5SD or TAT-ctrl (20 μM) for 24 h and cell death was measured by Trypan Blue dye exclusion. Three independent blood-draws were obtained on different days for CLL arbitrary patient numbers 1 and 2. **(D)** The average level of cell death induced by TAT-D5SD or TAT ctrl (20 μM) for 24 h in primary CLL cells from **(C)**. **(E)** TAT-D5SD peptide (20 μM) rapidly induces cell death in primary CLL cells for up to 24 h. **(F, left)** TAT-D5SD peptide induces apoptotic nuclear morphology in primary CLL cells. Shown are representative images of Hoechst staining of CLL cells following 4 h treatment with TAT-D5SD or TAT-ctrl (20 μM). **(F, right)** Quantification of apoptotic CLL cells assessed by Hoechst staining. **(G)** TAT-D5SD peptide induces PARP cleavage of primary CLL cells. Western blot showing PARP-1 cleavage in response to TAT-D5SD peptide vs TAT-ctrl (20 μM) following 4 h of treatment. **(H)** TAT-D5SD peptide-induced cell death is Ca^2+^-dependent. The MEC1 CLL-derived cell line was pretreated for 30 min with the intracellular Ca^2+^ chelator BAPTA-AM (10 μM) followed by treatment with TAT-D5SD or TAT-ctrl peptides (20 μM) for 2 h. **(I)** Representative single cell Ca^2+^ traces (≥140 cells) from MEC1 cells treated with TAT-D5SD peptide in the absence (left) or presence (right) of BAPTA-AM (20 μM). **(J)** Total effect of BAPTA-AM pretreatment on TAT-D5SD induced Ca^2+^ elevation shown in **(I)**, but quantified using a minimum of 140 cells. Cell death was measured by Trypan Blue dye exclusion unless otherwise indicated. Bars represent the mean ± SEM of triplicate measurements. Results were confirmed in multiple independent experiments. A student’s T-test was used to determine statistical significance; *P<0.05; **P<0.01; ***P<0.001.

CLL is a leukemia in which constitutive signaling through the BCR pathway is important to malignant B-cell survival (23, 24). While the expression of Lck varies among primary CLL samples (4, 9, 10), both Lck and Lyn were readily detectable by western blot analysis (Fig. 4A). Consistent with the MS analysis in Fig. 3F, Lck was detectable by western blotting in normal B cells when blots were exposed for longer periods of time. We also examined a database of 68 primary CLL samples and 103 B-cell lymphoma samples subjected to RNA-seq. **Fig S4** shows that 100% of CLL and B-cell lymphoma samples co-express Lck and Lyn. Additionally, we observed that Lck levels vary depending on how samples are prepared. For example, CLL6 showed lower levels of Lck protein when cell pellets were lysed in Ripa buffer (Fig. 4A), yet Lck was readily detected from a second blood-draw when cell pellets were lysed in concentrated SDS sample buffer (Fig. 4B). In the same sample, we found that both Y394 and Y505 sites were phosphorylated, suggesting that Src kinases are constitutively active in some CLL patients (Fig. 4B). This is also evident by the high level of tyrosine phosphorylation present in these cells.

In order to assess the effect of TAT-D5SD on CLL cells, we obtained peripheral blood from multiple CLL patients. Cells were immediately treated with either TAT-D5SD or TAT-control peptides for 24 h. Potent induction of cell death by TAT-D5SD peptide was detected in every sample tested (Fig. 4C). The average level of cell death in CLL patient samples was 52%, suggesting the IC_50_ of TAT-D5SD is ~ 20 μM (Fig. 4D). To evaluate the kinetics of cell death, TAT-D5SD and TAT-control peptides were evaluated at 3, 6, 9, 12, and 24 hrs. Cell death with TAT-D5SD peptide occured early (3 h) and gradually at 20 μM, whereas the TAT-control peptide had a minimal effect on cell viability (Fig. 4E). It was confirmed that the mechanism of cell death in CLL cells was apoptosis, which was evident by Hoechst 33342 dye staining of condensed nuclei 4–6 hours after treatment with TAT-D5SD peptide (Fig. 4F). This was further confirmed by PARP cleavage in a primary CLL sample 4 h after treatment with TAT-D5SD (Fig. 4G).

Because D5SD peptide binds to Lck and displaces it from IP_3_R-1, we hypothesized that this rapid induction of apoptosis was Ca^2+^-dependent. To test this, we treated the CLL-derived cell line MEC1 for 30 min with the intracellular Ca^2+^ chelator BAPTA-AM prior to incubation with TAT-D5SD peptide. As shown in Fig. 4H, the addition of BAPTA-AM prevented the induction of cell death by TAT-D5SD. Interestingly, when intracellular Ca^2+^ was measured by single cell digital imaging, TAT-D5SD induced Ca^2+^ mobilization into the cytosol within 10 minutes following the addition of peptide (Fig. 4I). As expected, Ca^2+^ responses induced by TAT-D5SD peptide were inhibited by the addition of BAPTA-AM (Figs. 4I and 4J). Together, these results suggest that the mechanism of cell death induced by TAT-D5SD in CLL is Ca^2+^-dependent.

### TAT-D5SD Peptide Induces Cell Death and Inhibits Proliferation in B Lymphoma Cells

**Figure 5:**
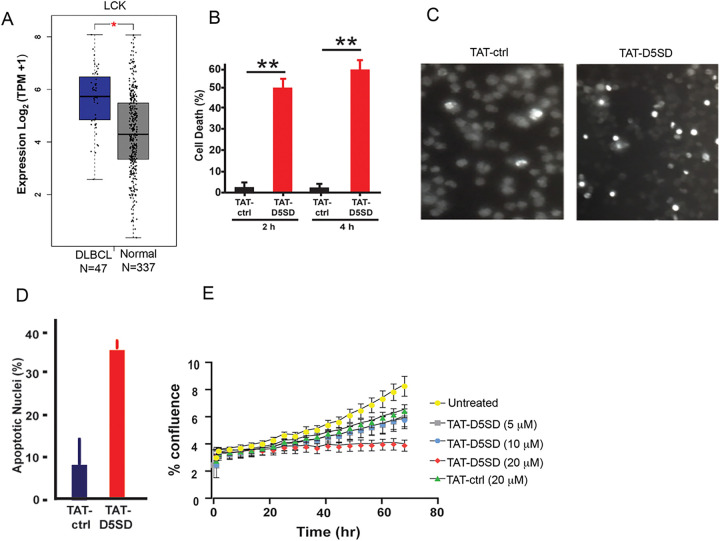
D5SD peptide induces cell death and inhibits cell proliferation in a model of DLBCL. **A)** *LCK*expression is elevated in DLBCL patient samples. RNA-seq data extracted from the TCGA database showing expression of *LCK* in DLBCL (N=47) vs matched normal controls (N=337). **(B)** TAT-D5SD peptide (20 μM) rapidly induces cell death in a diffuse large B-cell lymphoma (DLBCL) line, OCI-LY-10. Cell death was measured by Trypan Blue dye exclusion. **(C)** TAT-D5SD peptide induces apoptotic nuclear morphology in OCI-LY-10 cells. Shown are representative images of Hoechst staining of cells following 4 h treatment with TAT-D5SD or TAT-ctrl (20 μM). **(D)** Quantification of apoptotic OCI-LY-10 cells assessed by Hoechst staining after 6 h treatment. **(E)**TAT-D5SD peptide inhibits cell proliferation in OCI-LY-10 cells. NucLight Red-expressing cells were left untreated or treated with varying concentrations of TAT-D5SD or TAT-ctrl and cell confluence was assessed by live cell imaging fluorescence microscopy using Incucyte Zoom software based on phase-contrast images from 0 h to 72 h. Results for **(B)**through **(E)** were confirmed in multiple independent experiments. Bars and symbols represent the mean ± SEM of triplicate measurements. A student’s T-test was used to determine statistical significance; *P<0.05; **P<0.01; ***P<0.001.

Based on publicly available RNA-seq data from the Cancer Genome Atlas (TCFA), DLBCL is another B-cell malignancy that expresses *LCK* at significantly higher levels compared with matched normal cells (Fig. 5A). Interestingly, *LCK* showed a similar pattern of upregulation in DLBCL tumor samples as *BTK* and *SYK* **(Fig S5)**, both of which drive the BCR signaling pathway and are therapeutic targets in B-cell malignancies (25). To evaluate cell death induction in a model of DLBCL, we evaluated OCI-LY-10 cells which have previously been shown to express Lck (Table 1). Strikingly, a marked increase in cell death was observed after just a 2 h incubation with TAT-D5SD peptide (Fig. 5B). Apoptotic nuclear morphology was also apparent within this short time frame (4 h to 6 h) (Figs. 5C and 5D). We then subjected NucLight Red-expressing OCI-LY-10 cells to IncuCyte ZOOM live cell imaging fluorescence microscopy. This technique analyzes cell proliferation over time in a controlled environment tissue culture chamber without a need to disrupt cellular clumps (26). While sustained incubation of cells with TAT-ctrl peptide led to an increase in cell proliferation, cells treated with TAT-D5SD showed no proliferative capacity (Fig. 5E). These data suggest that TAT-D5SD peptide induces cell death in multiple types of B-cell lymphoma (see Table 1), including cell lines derived from more aggressive malignancies such as DLBCL.

### Lck Is Aberrantly Expressed in AML and Associates with Well-Characterized Oncogenes

**Figure 6:**
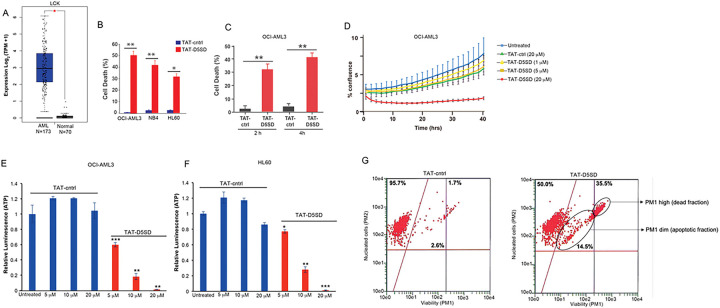
D5SD peptide induces cell death in AML cells. **(A)** *LCK* expression is elevated in AML patient samples. RNA-seq data extracted from the TCGA database showing expression of *LCK* in AML (N=173) vs normal controls (N=70). **(B)** Lck-expressing AML cell lines OCI-AML3, NB4, and HL60 were treated with TAT-D5SD or TAT-ctrl (20 μM) for 24 h and cell death was measured by Trypan Blue dye exclusion. **(C)** TAT-D5SD peptide (20 μM) rapidly induces cell death in AML cells. OCI-AML3 cells were treated with TAT-D5SD or TAT-ctrl (20 μM) for 2 h and 4 h. **(D)** D5SD peptide inhibits cell proliferation in OCI-AML3 cells. NucLight Red-expressing cells were left untreated or treated with varying concentrations of TAT-D5SD or TAT-ctrl peptides and cell confluence was assessed by live cell imaging fluorescence microscopy using Incucyte Zoom software based on phase-contrast images from 0 h to 40 h. **(E** and **F)** TAT-D5SD peptide diminishes the percentage of metabolically active AML cells. OCI-AML3 and HL-60 AML cell lines were treated with varying concentrations of TAT-D5SD or TAT-ctrl peptides and subjected to Cell Titer Glow (CTG) reagent to measure the presence of ATP. The level of ATP is proportional to the luminescent signal present in each well. **(G)** TAT-D5SD induces apoptosis in in OCI-AML3 cells. Cells were treated with TAT-D5SD or TAT-ctrl peptides (20 μM) for 24 h. Apoptosis was measured by ow cytometry using Guava ViaCount assay, which contains a proprietary combination of dyes that measure viable, dead, and apoptotic cell fractions. Cell death was measured by trypan blue dye exclusion unless otherwise indicated. Bars and symbols represent the mean ± SEM of triplicate measurements. Results were confirmed in at least three independent experiments. A student’s T-test was used to determine statistical significance; *P<0.05; **P<0.01; ***P<0.001.

Lck has been implicated as a potential driver of oncogenic transformation and cell proliferation in AML and was identified as a therapeutic target by the Gene Expression Omnibus database (15, 16). However, very little is known about the potential role of Lck in AML. While its expression in AML is low compared with lymphoid malignancies, RNA-seq data from TCGA shows that *LCK* is expressed in nearly all 173 AML samples analyzed by TCGA (Fig. 6A). Using computational approaches, we examined Lck for potential PPIs based on a number of AML-specific genes. A PPI network within the TCGA AML dataset is shown in **Fig S6A**. Interestingly, Lck is shown to interact with AML-specific oncogenes such as FLT3, Notch-1 and Kit. Indeed, the SH2 domain of Lck was previously shown to interact with FLT3-ITD in B cells (27), which suggests a role for Lck in FLT3-ITD positive AML. Intriguingly, the expression of the *LCK* gene in AML was similar to known drivers of AML leukemogenesis and proliferation including *FLT3, NOTCH1, KIT, RUNX1, RUNX2, DNMT3A, MN1,* and *CEBPA* **(Fig S6B)**. There were no differences in the expression of *LYN* and *SRC* between AML samples and matched normal controls.

### TAT-D5SD Peptide Induces Cell Death and Inhibits Proliferation in AML Cells

Given the potential importance of Lck in AML, we tested whether TAT-D5SD peptide would have activity in AML cells. Indeed, the AML cell lines reported in Table 1 displayed a high sensitivity toward TAT-D5SD peptide (Fig. 6B). TAT-D5SD peptide rapidly induced cell death in the AML cell line OCI-AML3 in 2 h (Fig. 6C). TAT-D5SD peptide dramatically inhibited cell proliferation when analyzed by live cell imaging (Fig. 6D). Additionally, AML cells heavily rely on cellular metabolism to proliferate. We observed that the level of metabolically active OCI-AML3 and HL-60 cells were significantly diminished with increasing concentrations of TAT-D5SD peptides (Figs. 6E and 6F), suggesting that the effects on AML cell metabolism are dose dependent. Last, we show that OCI-AML3 cells treated with TAT-D5SD undergo apoptosis after treatment with TAT-D5SD peptide. Flow cytometric analysis showed that half of the AML cell population was apoptotic at 24 hours and consisted of a dead (late apoptotic) fraction and viable (early apoptotic) fraction (Fig. 6G).

## Discussion

We set out to design an IP_3_R-1-derived peptide that would function as a competitive inhibitor to displace Lck. Using bioinformatic approaches, we predicted the region of domain 5 (within IP_3_R-1) that was facilitating the IP_3_R-1-Lck PPI. Specifically, we used the GOR IV method to target a region of domain 5 that was predicted to form alpha helices (Fig. 1B). Since Lck binds at Domain 5, the GOR method can be used to predict where the most alpha-helices, beta-sheets, and random coils are within the fragment (17). This approach is based on the principle that PPIs are more likely to occur in alpha-helical regions (28). Several drugs, including those that target p53/MDM2 and Bcl-2/Bax have been developed based on the identification of alpha helical regions (28).

Based on these predictions, a 21 amino acid region in the middle of amino acids 2078–2098 (KKRMDLVLELKNNASKLLLAI) was synthesized and referred to here as domain 5 sub-domain (D5SD) (Fig. 1A). Importantly, the two lysine residues at the N terminus of D5SD also facilitate PPIs, which was our rationale for choosing this specific alpha helical region. As a control, we designed and synthesized a scrambled peptide that had no significant homology to any mammalian protein.

Molecular modeling and pull-down experiments revealed that D5SD peptide can bind the SH2 domain of Lck in vitro (Figs. 1C and 1D). While SH2 domains typically interact with other proteins via phosphor-tyrosine, a tyrosine residue is not necessarily required for direct SH2 binding as is the case for Lck’s interaction with CD45 at the TCR (29, 30). We also showed that D5SD peptide binds to full-length Lck protein, whereas no physical interaction was observed between Lyn and IP_3_R-1 (Figs. 2A and **S1**). These data suggest that D5SD peptide may have preference for binding Lck over other Src family members.

One goal of this research was to provide a proof-of-concept that D5SD peptide could function as a novel therapeutic for a wide range of hematological malignancies. Importantly, the small basic protein TAT (86–101 residues) drastically enhances the efficiency of viral transcription and can be used to maximize delivery of both D5SD and control peptides into human cells (31). Given the rapid induction of cell death by TAT-D5SD, we hypothesize that the Lck-IP_3_R PPI may be important for the survival of leukemia and lymphoma cells. This is supported by the finding that cells deficient in Lck protein undergo a higher rate of apoptosis (Fig. 3B). In addition, selective targeting of the Lck SH2 domain by short phospho-tyrosine peptide is sufficient to induce cell death in CLL cells, as is pharmacological inhibition of Lck with dasatinib (Fig. 3C and 3D). We speculate that leukemic cells require a persistent Ca^2+^ flux via IP_3_R channels to positively regulate cell survival. When the Lck-IP_3_R-1 PPI is disrupted in T cells, the pattern of Ca^2+^ signaling is shifted after strong TCR activation (13). In CLL cells, cytoplasmic Ca^2+^ elevation occurs rapidly after exposure to TAT-D5SD peptide (Fig. 4I, left). The pattern of Ca^2^ elevation in lymphocytes can ultimately determine cell fate (32). Repetitive pulses that are shorter in amplitude and lower in Ca^2+^ concentration promote cell proliferation and survival, whereas larger Ca^2+^ waves that release more Ca^2+^ into the cytosol promote cell death. Figure 4H supports the hypothesis that the Ca^2+^ elevation is required for cell death. Given that Lck^hi^ cells are associated with enhanced BCR signaling (9, 10), it is possible that the Lck-IP_3_R PPI tightly regulates Ca^2+^ homeostasis, but in favor of cell proliferation and survival.

It is of particular interest that Lck is expressed in malignant cells derived from both lymphoid and myeloid origin. We among others have shown aberrant Lck expression in primary CLL cells (4–11). Using the publicly available TCGA database, we show that *LCK* is expressed and elevated in DLBCL relative to normal controls (Figs. 5A and S5). Of note, the pattern of *LCK* expression is similar to oncogenes *SYK* and *BTK*, which are known drivers of B-cell leukemia and lymphoma (25). While few studies have investigated Lck in myeloid cells, Lck has been shown to interact with the SH2 domain of FLT3-ITD and increases colony formation in pro-B cells (27). Considering the importance of FLT3-ITD in AML, we conducted a PPI network analysis using the TCGA AML dataset containing 173 human primary AML samples. This analysis shows Lck interacting with FLT3 as well as Notch-1 and Kit **(Fig S6)**. RNA-seq data reveals *LCK* is expressed in nearly all AML samples in Fig. 6A, and has previously been identified in AML by the gene expression omnibus database (16). In childhood AML, Lck clusters with other T cell proteins and was detected in 500 patient samples (33). In adult AML, Lck clusters with *NOTCH1, NOTCH3, CD74,* and *LGALS3* based on expression level, and these clusters are significantly associated with overall survival (data available at leukemiaproteinatlas.org). Moreover, dasatinib has shown to increase sensitivity in AML cells carrying FLT3-ITD (34), suggesting Lck could be a relevant therapeutic target in AML.

The discovery that TAT-D5SD peptide induces cell death in B-cell and myeloid malignancies, whereas T-cell leukemias and lymphomas had lower responses, is intriguing. The expression and cellular distribution of Lck may determine the relative sensitivity to TAT-D5SD peptide in lymphocytes. For example, B cells that express lower levels of Lck show increased sensitivity to Lck-IP_3_R-1 disruption by TAT-D5SD. However, a larger sample size would be needed to validate this correlation. This has important implications considering the high variability of Lck expression in B-cell malignancies. For example, CLL cells with high expression of Lck (Lck^hi^) have increased proliferative capacity and survival vs Lck^low^ cells (9, 10). While D5SD peptide does not physically interact with Lyn, it does bind other proteins such as PKM2 that interact with IP_3_R-3 (35), which could affect its cytotoxic potential in certain cell types. Importantly, we have shown that TAT-D5SD does not have cytotoxic activity in epithelial cells or fibroblasts; presumably this is because Lck is either not expressed or does not play a role in the survival of non-hematopoietic cells.

The induction of apoptosis by TAT-D5SD provides evidence that a peptide may have promise as a therapeutic agent for CLL, B-cell lymphomas, and AML. Peptide therapeutics are emerging in oncology due to their ability to be highly selective and cell-permeable (36). Novel therapeutic targets for AML are desperately needed, as the prognosis of patients is extremely poor, with median survival approaching one year (37). In the long term, we believe these data could be instrumental in developing and testing novel therapeutics that target the Lck-IP_3_R PPI.

## Materials And Methods

### Peptide synthesis

Peptides were synthesized by GenScript and purified by liquid chromatography/mass spectrometry to > 95% purity. The D5SD sequence is KKRMDLVLELKNNASKLLLAI. The control peptide sequence is NLNHSDQFAENLSHICGGHG. The TAT cell-penetrating peptide sequence (RKKRRQRRRGG) was added to the N-terminus of each peptide. The sequence for the SH2- binding phospho-peptide was EGQY*EEIP; the dephosphorylated peptide EGQYEEIP was used as a control.

### Antibodies and western blotting

The following antibodies were used in this study: Fyn (FYN3), Lck (3A5), and Lyn (44) (Santa Cruz Biotechnology); Phospho-Lck Y394 (Src Y416, 2101), Phospho-Lck Y505 (2751), Phospho-tyrosine (P-Tyr-100, 9411), Parp (9542) (Cell Signaling Technology); Actin (A-5441) (MilliporeSigma). Antibodies against IP_3_R-1 were kindly provided by Jan Parys and Humbert De Smedt (Rbt- 03) (Katholieke Universiteit Leuven) and by Richard Wojcikiewicz (CT-1) (SUNY Upstate Medical University). All western blots were performed as previously described (4). Raw uncropped western blots can be found in supplemental supporting information.

### Cell culture

All procedures followed the guidelines and regulations in accordance with IRB protocol ICC2902/11-02-28 of Case Western Reserve University Cancer Center/University Hospitals Cleveland Medical Center. The use of patient samples was approved by the IRB of Case Western Reserve University School of Medicine.

WEHI7.2 and HEK-293 cells were cultured in DMEM containing 10% FCS, 100 μM non-essential amino acids, and 2 mM L-glutamine. Jurkat, CEMC7, Raji, RS11846, MEC1, HL60, NB4, OCI-AML3, NL20, and NIH 3T3 cells were cultured in RPMI-1640 medium with 10% FBS, 100 μM non-essential amino acids, and 2 mM L-glutamine. OCI-LY-10 cells were cultured in IMDM with 20% FBS, 2 mM glutamine and 50 μM 2-mercaptoethanol. All cell lines were incubated in a humidified incubator at 37°C in 5% CO_2_ except for WEHI7.2 cells, which were incubated in 7% CO_2_. All cell lines except for WEHI7.2 and MEC1 were purchased from the American Type Culture Collection. The WEHI7.2 cell line was from University of California San Francisco and MEC1 from the DSMZ (Germany). Cell lines were routinely tested for mycoplasma.

### RNA interference

pLKO.1 lentiviral vectors with shRNAs targeting Lck or Fyn were transduced along with pMD2G (env) and pR8.74 (gag and pol) vectors into 293T cells to generate viral particles. Viral particles were subsequently incubated with WEHI7.2 cells in the presence of puromycin to positively select for transduced cells. Stably transduced, knock-down cells were assessed for Lck and Fyn expression by western blotting.

### Biotin-streptavidin pull-down assays

Biotin-labelled peptides were immobilized on streptavidin-coated beads and incubated with a biotin-containing buffer to block streptavidin molecules not bound to peptides. Beads were washed three times with Tris Buffered Saline (TBS; 25 mM Tris-HCl, 0.15 M NaCl, pH 7). Cell lysates, or puri ed GST-tagged SH2-Lck, were incubated with immobilized peptides for 18 h at 4°C. Beads were washed extensively and incubated for 5 min with 50 μL elution buffer prior to centrifugation. Protein analyte including eluate, beads, washes, and flow-through were analyzed by western blotting. The puri ed GST-tagged SH2-Lck domain was visualized by Coomassie Brilliant Blue staining on an SDS gel.

### Co-immunoprecipitation

Cells were washed in cold PBS and pellets were resuspended in Tris (50 mM), NaCl (100 mM), EDTA (2 mM), CHAPS (1%), NaF (50 mM), Na3VO4 (1 mM), phenylmethylsulfonyl fluoride (1 mM) and protease/phosphatase inhibitor cocktails. When applicable, total protein was incubated with D5SD or control peptides for 1 h at 4°C prior to immunoprecipitation with anti-IP_3_R-1 antibody. Immunocomplexes were subsequently incubated with protein G-agarose beads, washed extensively with cold PBS and CHAPS lysis buffer followed by denaturation and boiling in SDS sample buffer.

### Cell viability and apoptosis

Cell viability was quantified by Trypan Blue dye exclusion. Apoptotic nuclei were visualized and quantified by Hoechst 33342 dye using an Axiovert S100 Flfluorescence Microscope (Zeiss) equipped with a 40× oil objective (Zeiss) with excitation/emission at 350/535 nm. OCI-AML3 cells were subjected to Guava ViaCount (Millipore Sigma) to assess viable, dead, and apoptotic cell fractions and analyzed by flow cytometry. WEHI7.2 cells were subjected to dual staining with annexin-V and propidium iodide as previously described (4).

### Measurement of metabolically active cells

Metabolically active cells were assessed using CTG reagent (Cell Titer Glo). CTG was added to each well in a 96-well plate after a 24 h treatment and 10 min incubation at room temperature. Brie y, CTG reagent uses ATP as an indicator of metabolically active cells. The enzyme luciferase acts on luciferin in the presence of Mg^2+^ and ATP to produce oxyluciferin and to release energy in the form of luminescence.

### Measurement of cell proliferation

IncuCyte ZOOM (Essen Biosciences) was used as a measure of cell proliferation. NucLight Red-expressing cells were cultured in 96-well plates and phase-contrast images were taken every 2 h to determine cell confluency.

### Measurement of intracellular Ca^2+^

Cells were pre-treated with the intracellular Ca^2+^ chelator BAPTA-AM (10 μM) for 30 min and single cell Ca^2+^ traces were recorded in real-time with a Zeiss axiovert S100 microscope (Carl Zeiss AG). Excitation wavelengths were programmed to alternate at 340 and 380 nm at 1 s intervals to monitor changes in intracellular Ca^2+^ concentration in fura-2 loaded cells as previously described (38).

### Prediction of secondary structure

The GOR IV algorithm was used to predict the secondary structure of Domain 5 within the IP_3_R protein sequence (17). A region of domain 5 was chosen based on the high probability of alpha-helices vs other secondary structure elements. Output of the GOR IV algorithm was obtained using ALIGNSEC, a computational module that is part of ANTHEPROT package and available at http://antheprot-pbil.ibcp.fr (39).

### Molecular modeling

Molecular modeling was performed using GalaxyPepDock software on an interactive web server (40). Brie y, the modeling algorithm is based on a flexible structure energy-based approach. Using previously identified structures within the protein data bank (PDB), input peptide sequences are aligned for similarities in molecular structure to calculate both a similarity and interaction score. The structural interaction analysis generates a number of templates which are subjected to energy optimization. This process increases the accuracy of the modeling by sampling backbone and side-chain flexibilities for both the protein and peptide. After each model is refined by energy optimization, the output containing up to 10 predictive models is generated. GalaxyPepDock is freely available at http://galaxy.seoklab.org.

### PPI network analysis

PPI networks were generated using the Protein Interaction Network Analysis (PINA version 3.0) (41). Brie y, this program integrates data from multiple protein databases (IntAct version 4.2.15, BioGRID version 3.5.185, MINT May 21, 2020, DIP version 20170205, and HPRD release 9) and generates a non-redundant protein interactome that aligns with RNAseq expression data from the TCGA AML dataset containing 173 patients and 16,731 genes. Each node represents a potential PPI with the query protein. Nodes are color-coded based on tumor-specific expression and survival data. Edges connecting each node represent the correlation between the query protein and the interacting protein. The relative width of each edge designates the strength of the correlation (Pearson R^2^ correlation coefficient). PINA is freely available at https://omics.bjcancer.org/pina/.

### Genomic and proteomic data mining

Heat maps and scatterplots of expression data were obtained using GEPIA2, a computational web server for large-scale expression pro ling and interactive analysis (42), and the EMBL-EBI expression atlas (43). GEPIA2 analyzes RNAseq data from 9,736 tumors and 8,587 normal samples from the TCGA database. In this study, we utilized AML data from 173 patients with matched normal controls and DLBCL data from 47 patients and matched normal controls. GEPIA2 is freely available at http://gepia2.cancer-pku.cn/. High resolution Fourier mass spectrometry data was obtained from the human proteome map (44) and visualized with the EMBL-EBI expression atlas (43).

### Experimental reproducibility and statistical analysis

Data are presented as the mean ± SD or SEM as appropriate; a minimum of three measurements were obtained from at least three independent experiments. A Student’s t test was used to determine statistical significance between two treatment groups. A two-tailed p-value of 0.05 was the minimal threshold for significance.

## Figures and Tables

**Table 1:** Cell lines used in this study

Cell line	Description	Species	Lck detection by western blot analysis	Reference Citation
**WEHI7.2**	T-cell lymphoma	Mouse	**Yes** (Harr et al, 2010)	(4)
**Jurkat**	T-cell ALL	Human	**Yes** (Lo et al, 2018)	(45)
**CEMC7**	T-cell ALL	Human	**Yes** (Harr et al, 2010)	(4)
**MEC1**	Chronic lymphocytic leukemia	Human	**Yes** (Talab et al, 2013)	(9)
**OCI-LY-10**	Diffuse large B-cell lymphoma	Human	**Yes** (Ke et al, 2009)	(46)
**Raji**	Burkitt lymphoma	Human	**Yes** (Deans et al, 1995)	(47)
**RS11846**	Follicular lymphoma	Human	**Yes (** Fig. 3 **)**	– – – –
**OCI-AML3**	Acute myeloid leukemia	Human	**Yes** (Fiegl et al, 2009)	(48)
**HL60**	Acute myeloid leukemia	Human	**Yes** (Kropf et al, 2010)	(49)
**NB4**	Acute myeloid leukemia	Human	**Yes** (Kropf et al, 2010)	(49)
**HEK-293**	Embryonic kidney cells	Human	**No** (Lo et al, 2018)	(45)
**NL20**	Bronchial epithelial cells	Human	**Yes** (Rupniewska et al, 2018)	(50)
**NIH 3T3**	Embryonic fibroblasts	Mouse	**No** (Gervais et al, 1997)	(51)

Cell lines used in this study listed by disease, species of origin, and whether or not Lck expression was detected by western blotting.
